# Fear, “Discomfort,” Anger, and Shame in the Night-Time Economy: Women's Responses to Unwanted Sexual Intrusions

**DOI:** 10.1177/10778012241259719

**Published:** 2024-06-07

**Authors:** Ruth Lewis, Amanda McBride

**Affiliations:** 1 Department of Social Sciences, Northumbria University, Newcastle upon Tyne, UK; 2 Department of Social Work, Education and Community Well-being, Northumbria University, Newcastle upon Tyne, UK

**Keywords:** gender-based violence, night-time economy, sexual harassment, emotions

## Abstract

Despite a surge of attention to gender-based violence (GBV), research about the night-time economy (NTE) as a site of gendered violence is limited. Even less research examines women's emotional responses to “unwanted sexual intrusions” (USI) in the NTE. Analyzing women's emotional responses can generate deeper understanding of social phenomena, power and its operation, and is in keeping with feminist theorizing that uses a victim-survivor-centered approach. Analysis of qualitative data, from a survey we conducted in the United Kingdom, reveals women experience USI in the NTE as a frightening, shameful injustice. The article discusses these emotions in light of the changing “emotional climate” about GBV.

## Introduction

Despite current unprecedented levels of attention to the topic of men's violence against women (VAW), the night-time economy (NTE) has been relatively neglected in scholarly research, even though it is a public space where sexual violence is pervasive. Even less research examines women's emotional responses to sexual violence in the NTE. This article addresses that gap by analyzing survey data that reveal women experience unwanted sexual intrusions as a frightening, shameful, “uncomfortable” injustice.

The behaviors that are the subject of this article have been variously named as “sexual harassment,” “unwanted sexual attention” (Fileborn, 2012), “intimate intrusions” (Stanko, 2013), or by women themselves, as “hassle” or “creepiness.” The range and variety of behaviors, as well as their normalization which can make them invisible to many, mean it is difficult to find a satisfactory term that both adequately describes these behaviors and indicates the motives behind them (Vera-Gray, 2016). Kelly's (1987) concept of the “continuum” of sexual violence is invaluable in demonstrating that the variety of behaviors, ranging from the moderately uncomfortable to the criminal, are connected theoretically because they are underpinned by and reinforce gendered power hierarchies. We use the term “unwanted sexual intrusions” to refer to this range of women's experiences in the NTE. We construct this term by borrowing from Fileborn's (2012) “unwanted sexual attention” and Stanko's and Vera-Gray's focus on “intrusions” (Stanko, 2013: “intimate intrusions”; Vera-Gray, 2016: “men's stranger intrusions.”) The NTE is a space where sexual attention may be sought and consensually exchanged so it is important to distinguish desired from “unwanted” attention. However, in reviewing the data we discuss in the article, we note that although the term “attention” accurately described some women's experiences, it does not convey the invasive nature of other experiences, the “deliberate act of putting oneself into a place or situation where one is uninvited, with disruptive effect” (Vera-Gray, 2016, p. 15). The term “intrusions” seems more fitting to describe situations when men persistently intrude on a woman's space despite her protestations, and/or when they physically assault women, often on parts of their bodies considered “intimate” (such as breasts, bottoms, groins). Whatever the nature of the act—staring, verbal comments, or physical assault—these unwanted sexual behaviors intrude on the freedom and selfhood of the person targeted. Therefore, we offer the term “unwanted sexual intrusions” (hereafter, USI) to describe the wide range of women's experiences of this type of violation.

USI are gendered, not only because they are usually (but not only) perpetrated by men against women (Bows & Fileborn, 2022), but also because the contexts and consequences are gendered. Women's experiences of USI occur in the wider context of men's (lethal and non-lethal) VAW in the home, in public spaces, at work, as well as in the context of a wider discourse about VAW (e.g., in films, TV, computer games, and the media); together they form a continuum (Kelly, 1987). Girls are primed, from a young age, to be alert to and to avoid the risk of men's violence and consider themselves “lucky” if they manage to avoid it (Fileborn, 2016). With this backdrop, encounters with USI can trigger fears of further violence, including sexual and lethal violence. Men, as staff and customers of the NTE, can also experience USI, perpetrated by men or by women. A key gendered difference between men's and women's experiences is that men's victimization does not occur in a wider context of sexual violence so fear of further consequences, including sexual and lethal violence, is less likely to be part of men's experience. Lesbian, gay, bisexual, transgender, and queer (LGBTQ) people are also the targets of USI, from both within and outside the LGBTQ community (Fileborn, 2016); the forms can include hyper-sexualization and fetishization of LGBT people. This article's focus is on women's experiences of USI in the NTE, drawing on survey data.

In acknowledging the scope and diversity of USI, we must also consider how best to frame those who carry out such intrusions. While the term “perpetrator” is common in literature about gender-based violence (GBV), and perhaps best captures the intentionality of those who intrude, it also has a legalistic resonance and is out of step with, not only the reality of USI (much of which is not criminal), but also the terms used by women whose experiences we are bringing to the fore. So, instead, we use the terms “sexual intruders” or “men who sexually intrude” to highlight the ways in which their actions, regardless of intent, cross spatial, and personal boundaries. As we go on to argue, these intrusions are harmful, not only in the sense that individual women may be harmed by their occurrence, but also in that they have the effect of curtailing women's use of space and in doing so maintain the unequal power relations from which other gendered harms result.

In the following section, we explore the relevant existing scholarship about how women respond to USI and identify the relative dearth of attention to women's emotional responses. We also review relevant scholarship about the sociology of emotions to help us understand women's emotional responses. Data about women's emotional and behavioral responses to USI gathered from a survey conducted in the United Kingdom are presented; they demonstrate the affect associated with USI and the obstacles women face as they try to challenge the men who sexually intrude and to protect themselves. The article then goes on to explore the social nature of women's emotional responses which may reflect the changing public conversations about GBV.

## Understanding Responses to USI in the NTE

As with other forms of GBV, victim/survivors’ experiences of USIs have been overlooked, normalized, and trivialized. Although the NTE is a highly regulated space (Hadfield et al., 2009), regulation tends to be directed at alcohol and drug consumption and “anti-social behavior”—generally interpreted as men's violence to each other—rather than focusing on USI of women. A result is that our focus—USI—often falls through the gaps of this regulatory infrastructure, until it meets the threshold for criminal activity (and even then it rarely receives attention from the criminal justice system). Therefore, the burden of responding to USI typically falls to women and so, we argue, it is valuable to study their responses.

The relatively sparse literature about USI in the NTE, usually based on small samples of students, considers some elements of how women respond and how they manage risk. Although women's responses to USI include emotions and behaviors, previous literature rarely distinguishes between these and, while previous research has *referred* to women's emotional reactions to USI, few studies analyze this aspect of their responses. This may limit understanding of the nature of the phenomenon and its impacts for reasons we outline later on. Graham et al. (2017) find that emotional reactions to USI range from not taking it seriously or feeling flattered to, more commonly, feeling annoyed, uncomfortable, disrespected, violated, disgusted, angry, embarrassed, humiliated, and/or afraid. Diaz-Fernandez and Evans’ (2020) study about the affective dimensions of lad culture refers, almost in passing, to women's anger, resentment, and resignation in response to USI. Young women in Brooks’ (2014) study about a particular form of GBV—drink spiking—expressed uncertainty about whether they had been spiked and minimized their experiences, blamed themselves, and believed that their experiences would not be taken seriously if they sought help from venue staff, a belief supported by Fileborn's (2017) findings.

In contrast to limited scholarly attention to *emotional* reactions, more research focuses on reactive *behaviors* by women when they experience USI in the NTE. These behaviors range from carefully and gently communicating lack of interest while trying to avoid an aggressive reaction from the man, direct refusal, evasion, and avoidance (e.g., moving around a venue to try to lose a man), giving “dirty looks,” flashing one's engagement ring, claiming one has a boyfriend or is lesbian, invoking a friend's intervention, to using some form of physical resistance (e.g., digging in their back, stepping on their foot, pushing away, using or threatening to use shoes as weapons; Diaz-Fernandez & Evans, 2020; Fileborn, 2012; Graham et al., 2017; Gunby et al., 2020). Gunby et al. (2020, p. 36) delineate the “complex” and “diplomatic” emotion work women invest in these behaviors, especially in ending encounters with men who show sexual interest in them, without being subjected to abuse from the man in response to the rejection.

Women's reactive emotions and behaviors are connected, but not always in straight-forward ways. Graham et al. (2017) found that women who experience stronger negative emotions were more likely to behave more assertively by responding directly, aggressively, or by leaving the premises. However, while feeling afraid was also related to responding aggressively, “the effect was smaller than for most other feelings, perhaps reflecting that fear might prevent, as well as provoke, an aggressive response” (Graham et al., 2017, pp. 1434–1435). Fear can provoke a range of actions, depending on the assessment of the context and likelihood of escape or escalating violence. Challenging the traditional male-centric depiction of “instincts” as “flight or fight,” Rape Crisis England and Wales (no date) describes the five automatic, instinctive responses to fear—freeze, flop, friend, fight, or flight. Responses to USIs, as with threats more broadly, combine a mixture of instinctive and subconscious, as well as conscious and deliberate behaviors.

Some emotional and behavioral responses to USI are influenced by gendered socialization which communicates norms and expectations of girls and women (as well as boys and men). Fileborn (2016) notes that “[w]omen are inculcated from a young age to assume responsibility for the prevention of sexual violence” (p. 1107). This is reflected in almost every aspect of women's lives including, in the NTE, how they dress, hold themselves, walk, engage (or not) with men and other women, consume alcohol, and make choices about accessing public and private spaces and transport. These behaviors are experienced as a mixture of conscious and subconscious acts as they try to make sense of their experiences (Anitha et al., 2021). Research shows that, in the NTE, young women adopt strategies such as staying with people with whom they feel safe and being selective about the kinds of venues they attend (Fileborn, 2016), protecting their drink to avoid spiking (Brooks, 2014), and “dulling it down” by not dressing in overtly sexualized ways or by dressing in androgynous ways (Nicholls, 2017, p. 260). The range of behaviors and routines that young women adopt “become a normalised, embodied act and a means of self-governance” (Fileborn, 2016, p. 1108). Nicholls refers to these behaviors as “‘doing’ heterosexual femininity” (2017, p. 262).

Paying attention to the ways in which young women negotiate the risks of USI in the NTE highlights the myriad forms of resistance enacted in everyday—or night—spaces. Anitha et al. (2021, p. 2059) argue that some of these strategies, such as claiming to have a boyfriend or claiming to be lesbian, “reinscribe the gendered norms that underpin the behaviors and attitudes that they seek to evade.” However, they note their participants’ frustration with the inefficacy of many strategies of resistance and argue that women's strategies are limited by “the cultural context of the NTE … [as well as] by broader gendered sexual scripts” (Anitha et al., 2021, p. 2058).

Managing risks of and responding to USI is such an intrinsic part of women's experiences of the NTE that it is deeply implicated in the production of gendered identities; for Fileborn (2016, p. 1109) “young adults perform their gendered identities ‘*in and through*’ the production of safety in pubs and clubs” (italics in original). Gunby et al. (2020) define a particular form of gendered identity as “feisty femininity.” Drawing on accounts of their participants (student lawyers at an English university) which describe their “angry and overtly retaliatory responses” (p. 39) to USI in the NTE, Gunby et al. (2020) identify femininities that are assertive, “sassy,” and independent, with feminist undertones that challenge hegemonic masculinity, inspired, perhaps, by the recent visibility of feminism and feminist resistance to men's violence. While Gunby et al. (2020) argue, optimistically, that this form of femininity “will have a role to play in efforts aimed at eradicating gendered violence,” they also note that some of their participants who enacted these “feisty” behaviors had mixed feelings about them; they felt both entitled to challenge men's behaviors in this way but also worried about the consequences, presumably because of the risk of aggressive responses from men as well as judgments from other women. Indeed, in Fileborn's (2012) study some young women were critical of friends who responded to USI in ways seen to escalate the risk of violence from men. While women may consider themselves “as citizens who are without questioning entitled to protection from adverse sexual experiences” as Honkatukia et al. (2023, p. 1395) found in their study of young people in Helsinki, Finland, they may still be constrained in their responses to USI.

Moreover, while an assertive, empowered form of femininity may be welcomed, there are concerns (acknowledged by Gunby et al., 2020) that this “feisty femininity” reflects well-established discourses that hold women responsible for their own victimization. Rooted in patriarchal notions of victim blaming, these discourses have been re-energized by neoliberal, postfeminist imperatives for women to be responsible agents, unbowed by structural forces. In the NTE, “feisty femininity” underscores the absence of responses from others—such as venue staff, security personnel, and bystanders—and, arguably, reinscribes responsibility for responding to USI with those who are intruded upon. Moreover, as our data demonstrate, women experience myriad emotions and behaviors in reaction to USI, not all of which enable a “feisty” response. Furthermore, the immediate environment in which the USI is perpetrated may preclude such a response because of the risks associated with challenging perpetrators if one is alone or not with supportive allies. This raises the question of whether a “feisty” response *works*; while it might relieve frustration, and resist victimization or objectification, it does not necessarily prevent (further) USI.

The scholarship reviewed above tends not to linger on women's emotional reactions and focuses instead on how women negotiate and behave in response to USI. There is a sense in which their emotional reactions are unexceptional and can be “taken for granted.” By contrast, we see value in examining the emotions provoked by USI for three reasons. Firstly, as Åhäll (2018, p. 38) notes in arguing for a feminist politics of emotion, studying emotions generates a deeper understanding of social phenomena because “how we feel (consciously or unconsciously) about the world already tells us about how the world works.” These feelings shed light on experiences of power and its operation, on the “hidden injuries” (Sennett & Cobb, 1972) it generates, as well as on the material and discursive contexts of social phenomena, such as USI. Discourse is not only about words but also about “affective logic and practice” (Åhäll, 2018, p. 3) so to understand—and change—discourses, such as those about USI, requires attention to their emotional dimensions. Secondly, sociologists and feminists are interested in (changing) social phenomena, power, and power dynamics, none of which operate in an emotion-free vacuum. The experience of power—of having or not having power, of coming up against power and being reminded of one's relative powerlessness—is never experienced through cognitions and action alone; it is always experienced emotionally too. Finally, feminist approaches to examining social phenomena such as GBV have adopted a victim-centered approach which take seriously women's emotional (as well as cognitive, cultural, and material) experiences and insights. Indeed, attention to the affect associated with a phenomenon reveals the layers of meaning that are attached to that phenomenon. It also shows that individuals bring their own subjective understandings, based on their biographies, along with their socially patterned understandings that stem from their social positioning. Emotions are not (only) spontaneous and “of the moment”; they are also the result of “temporal layering” (Mason, 2018, p. 178), as memories and feelings—including those relating to experiences of gendered abuse—are accumulated and laid down throughout one's personal history. This victim-centered approach has generated important new insights and theories. For example, Kelly's (1987) “continuum of sexual violence” helped name and theorize women's subjective perceptions and the commonalities in their experiences as both a form and consequence of men's oppression of women.

Sociological analysis of emotions has forefronted the *social* nature of emotions. Bericat (2016, p. 495) notes that most emotions “emerge, are experienced and have meaning in the context of social relations.” In particular, the four fundamental emotions—fear, anger, shame, and pride—“are explained not so much by their importance in individuals’ psychic lives, but by the fundamental role they play in the social structure and in social dynamics” (Kemper cited in Bericat, 2016, pp. 501–502). According to Kemper's structural theory of emotions, emotions can be understood as the result of two dimensions of social interaction—power and status. Social interactions experienced by individuals with relatively low levels of power can generate feelings of fear; interactions experienced by those with relatively low levels of status can generate feelings of shame. These feelings are not just individual experiences, they are social in the sense that they are the result of social interactions between people in different positions in social structures, as well as in the sense that they can be group emotions, shared by members of the same group. The gendered and sexual dynamics at play in the NTE—a highly gendered and sexualized space—shape the social interactions and the emotional dimensions of these interactions. Our examination of these emotional dimensions focuses less on the micro-interactions and more on the macro social emotions to illuminate the nature, impact, and function of USI in the contemporary gender regime.

## Methods

The data presented in this article stem from an online survey conducted in the UK in autumn, 2021. The survey was launched in collaboration with Rape Crisis Tyneside and Northumberland (RCTN) and their social marketing partner, Crystallized. RCTN and Crystallized planned to conduct a survey in Newcastle upon Tyne to ascertain familiarity with the Shout Up! brand (https://www.shoutup.org/), an initiative launched by these organizations and funded by the local council to upskill venue and bar staff to identify sexual harassment and train them to intervene safely. The second author of this article was employed by RCTN to deliver the Shout Up! initiative and so the opportunity to collaborate on the survey emerged. A survey was selected as a data-collection method as it was the most efficient way of engaging with a sizeable, diverse sample of people who engage in the NTE. The survey was designed to be simple and short. It invited respondents to “tell us about your experiences of sexual harassment in the night-time economy” followed by the statement: “Feel free to tell us about all your experiences or chose one to tell us about. We’re particularly interested in what happened, the sex/gender of the harassers, what you or anyone else did in response to the harassment, how it made you feel at the time, how it made you feel afterwards and what impact/s (if any) it had on you.” Definitions of “sexual harassment” and “night-time economy” were provided^1^ and closed questions gathered data about demographics and where in the UK the harassment occurred.

The purpose of designing such a simple, short, unstructured questionnaire was to boost participation. This inevitably limited the extent to which we could ask for more detailed information about respondents’ experiences; future research could attend to this. We rely on respondents’ accounts of their emotions reported after the events that provoked them. In recalling the event and its impact, some emotions may have become crystallized and others may have diminished. Respondents' recall may have been shaped by reactions from others when they shared their stories (if they did) and by wider cultural and social events (such as publicity about other incidents of USI and/or other types of GBV). Other methods (such as diaries, interviews, and ethnography) might be useful for eliciting more detail and more timely accounts of feelings surrounding USI. However, many of the respondents provided detailed accounts of their experiences; responses ranged from 4 to 482 words. About half of the respondents gave information about their feelings in relation to the USI. Clearly even a simple question can effectively elicit rich data about experiences and emotional reactions.

The link to the survey was circulated amongst the authors’, RCTN's and Crystallized's networks and recipients were encouraged to share it with their contacts. In addition, it was promoted alongside other RCTN and Crystallized campaigns for a period of about 3 months. In total, 215 valid responses were generated. Most respondents were women; five were men (only three of whom reported their own experiences of USI), four nonbinary, and three “preferred not to say.” The answers of these 12 respondents have been excluded from the analysis for this article as they are small minorities in the dataset who, because of their sex or gender identity, might have quite different experiences of USI in the NTE. Table 1 describes the demographic data of the sample of 203 women.

**Table 1.** Respondents’ Age, Ethnicity, and Region.

		Number	As percentage of those who gave info
Age *n* = 151			
	18–25 years	73	46
	26–35 years	54	34
	36–45 years	26	16
	46+ years	7	4
Ethnicity *n* = 193			
	White	168	83^a^
	Asian/British Asian	15	7
	Mixed or multiple ethnic backgrounds	9	4
	Black/African/Caribbean/Black British	8	4
	Other ethnic group	3	1
Region *n* = 197			
	North East	78	38
	North West	74	36
	Scotland	31	15
	Elsewhere in UK	15	7
	Elsewhere in the world	5	2

a This broadly reflects the ethnic profile of the UK population (Gov.uk, no date, https://www.ethnicity-facts-figures.service.gov.uk/#:∼:text=Government%20data%20about%20the%20UK's,a%20variety%20of%20ethnic%20backgrounds).

The sex of those who sexually intrude was not revealed by all the respondents. The majority indicated the intruder(s) were men by referring to “man,” “guy,” or “lad,” or using male pronouns. Some did not indicate their sex. However, because it is widely understood that most people who perpetrate sexual intrusions are men, and because it would therefore be remarkable if the intruder were a woman, we think it is likely that respondents would mention if they experienced USI from by a woman. Indeed one woman (who is not included in the sample of 203 because she did not mention an emotion) did state that the person who intruded on her was a woman. Based on this rationale, we assume that, where the sex of intruders is not given, it is highly likely to be male.

### Analytical Approach

We started our analysis by reading the data multiple times to familiarize ourselves with it. As the project was open and exploratory, driven by a desire to examine what women said about their experiences of USI, we adopted an inductive approach and were alert to any patterns in the data that might indicate significant themes. It became clear that women's accounts of their responses to USI presented a fruitful line of inquiry, as it revealed their emotional reactions and was an understudied topic (as noted above). We created a secondary dataset comprised of every bit of data that mentioned emotions; we excluded data that *suggested* an emotional reaction but did not explicitly mention an emotion, as we were interested in respondents’ self-declared emotions. “Freezing” or being “numb” were not categorized as emotions because we interpreted these as behavioral responses which did not explicitly reveal respondents’ associated range of feelings. Through familiarizing ourselves with the secondary dataset and discussing the differences and similarities in the emotions expressed, we identified a set of 10 clusters which encapsulate the full range of emotions expressed by the respondents (see Table 2). We allocated each cluster a generic term (e.g., “fear,” “anger,” and “discomfort”) and, in the table, also presented the terms used by women.

**Table 2.** Women's Reports of Emotions in Response to USI.

Emotion expressed by women (our generic term in bold, followed by terms used by respondents)	Number of women expressing emotion^a^
**Fear**—scared, wary, unsafe, anxious, intimidated, on edge, petrified, threatened, terrified, uneasy, nervous, cautious, on high alert, insecure	37
**Discomfort**—uncomfortable	20
**Anger**—angry, annoyed, infuriated, hate it	19
**Upset**—upset, horrible, traumatic, awful, hurt, confused	14
**Vulnerable**—vulnerable, violated, objectified, small, insecure, invalid, powerless	11
**Shame**—embarrassed, mortified, guilty, ashamed, my fault, humiliated	10
**Disgust**—disgusted, sickening, horrible	7
**Shock**—shocked, startled, shaken	7
**No emotion**—desensitized	1
Total	126

a Eighty-four women expressed an emotion, some women expressed more than 1.

It is important to note that frequency of expression of certain emotions might not indicate simply that those emotions are most significant. It might instead indicate that these are normative emotions for women to express about USI. In addition, the anonymity and noninteractive nature of completing a survey may enable women to express emotions which they might not express in other interactions. Although the survey did not facilitate exploration with respondents of the potency or durability of their feelings, there were many—28—emphasizing words like “so” and “very” giving some indication of their intensity. Below, we present the findings as a reflection of personally-felt affect as well as socially normative emotions about USI at this point in the early twenty-first century. Data are anonymized to avoid revealing identifying data such as the names of cities or venues in the NTE.

## Findings*:* Women's Emotional Responses to USI in the NTE

USI manifests in a wide variety of behaviors; a detailed analysis of the types of USI is beyond the scope of this article but, in presenting data about women's accounts of their responses, some of this variety is revealed. In this section, using the survey data, we explore the range of emotional reactions.

USI provokes a variety of emotions, many of them experienced intensely and as embodied feelings. Eighty-four women in the sample group mentioned at least one emotion, some mentioned several, and emotions were mentioned 126 times. Table 2 shows the emotions and how many of our respondents expressed each one. Out of these 84 respondents, only 1 described feeling desensitized: “The thing is, I wasn’t even bothered by it for a long time, because it happens so often I was almost desensitized by it happening.” Some others also minimized the significance of their experiences:

Most nights out men will touch me inappropriately without my permission at nightclubs, I feel disgusted and angry [at] the time, afterwards I’m not too bothered as it's not too bad of sexual harassment.^2^

These responses which compare women's experiences with those of others, are significant because they show the ubiquity of USI; the respondents are able to compare their own experiences with those of others because they know that USI is so common. In addition, the rationalization of their experiences on the grounds that USI happens “most nights” points to its normalization, as we discuss below. Comparisons with “worse” experiences reflect an internalized logic to manage emotion which is likely influenced by societal norms about the “right amount of panic” (Vera-Gray, 2018). They may also reflect women's uncertainty about whether they can expect recognition of their experiences or, perhaps, their reluctance to accept the status of “victim.”

### Fear

In contrast to these responses, the predominant emotion expressed was fear, which Pryor et al. (2023) call an “often overlooked aspect of sexual violence” despite its ubiquity:

Well creepy men follow me and my friends and it scares us all and we always have to run.

We went to a night out and my friend was harassed by a drunk man. He followed us to the uber [taxi] and I was so scared. I even reported to the student campus.

There has also been an instant where I was potentially spiked in a club and become paralytic. This was very scary in retrospect as it wasn’t known how I got home, there were strangers with me. I had lots of nightmares and anxiety about it in the week following.

Bericat (2016) sees fear as part of an emotional family composed of worry, anxiety, panic, terror, and horror. Women's expressions of their fearful emotions ranged from feeling “anxious,” “uneasy,” and “scared,” to feeling “intimidated,” “terrified,” and “petrified.” Women's sense of fear was sometimes expressed in terms of lack of safety or as “threatening”:

Male forcing himself upon me and my female friends whilst being drunk. Felt threatening and his behavior wasn’t welcome. Felt like we had to leave the club but felt like he could follow us.

A lad about my age followed me home from a night out and wouldn’t stop tryna [trying to] talk to me, I told him I didn’t want to talk and he started shouting at me a[nd] saying I was a cunt and thought I was too good for him. I was scared and angry.

While there is no consensus among scholars, there is some agreement that fear, along with anger and shame, are primary emotions because they are “connected in the same way to social relational outcomes in all social categories and groups” (Kemper, 2006, p. 110). That is, we might consider them to be universal emotions rather than culturally varying. In his power-status theory of emotions, Kemper (2006) theorizes that fear (and anxiety) results from the power (rather than status) dimension of social relations. Fear is the felt emotion when one senses that one's own power is insufficient in an encounter. The accounts women provided suggest that their fear stems from anticipation of negative actions by the intrusive men. In their accounts, the anticipation is implicit, as if it is obvious that they fear further (sexual) violence. Instead of naming what they fear they name the preventative action they took—“we always have to run”; “I even reported to the student campus”—or the risks posed by strangers—“there were strangers with me.” In these accounts, sexual violence is “unspeakable” (Fileborn, 2019); it is both not necessary to mention it because it is obvious, but also not bearable to mention it because it is so fearful.

The emotion of fear in these contexts is both an individual and a social, collective emotion. It can be considered social in that it is a result of a social interaction, as well as in that it is a result of culture; girls and women learn, through socialization around “feeling rules” (Hochschild, 1979), to fear sexual (and nonsexual) violence from men, especially men who are “strangers” (Pryor et al., 2023). Women and girls as a collective learn that fearfulness is an appropriate feminine emotion. We understand the feelings of fear expressed by women as resulting from anticipation of (sexual) violence which is both an individual and a collective experience.

### Discomfort

Many respondents used the term “uncomfortable” to describe their emotional reactions. This is an elastic term that has come to cover a range of emotions, so it is difficult to determine its meaning, but some examples from the data give an indication of the types of behaviors women connect with “discomfort”:

An old man was trying to dance behind me but he could tell I was uncomfortable.

A bunch of men in a pub wouldn’t leave my friend and I alone and we felt very uncomfortable.

Males interrupting our conversations with chat up lines, ignoring our polite requests to move on, being inappropriate and making us uncomfortable.

The dictionary definition of “uncomfortable” is “uneasy” or “awkward” (Soanes & Stevenson, 2008). These seem relatively mild emotions, so it is surprising to see this term used to describe physical assaults such as:

Groped multiple times on nights out by men, top pulled down—not massive impact to me but was still an uncomfortable experience.

• on a night out with 3 girl friends • two males followed us everywhere, toilets, bar outside • one put his hand up my skirt and grabbed my chest • felt uncomfortable • don’t do [go] out anymore.

Men squeezing your bum when in a packed but [pub] or bar, staring and making you feel uncomfortable.

How can we make sense of the use of this term to refer to such intrusive behaviors which include criminal acts? Our respondents’ use of the term “uncomfortable” may reflect the “normalization” of GBV, whereby USI has become an expected, inevitable feature of a night out, unremarkable in its ubiquity (notwithstanding some women's rejection of such norms). Its normalization operates to brand those who challenge or draw attention to USI, “killjoys” (Ahmed, 2010) who “can’t take the heat” of a night out, and it may encourage victim-survivors to downplay the behavior and its impacts in order to preserve their own identity as a fun-seeking participant in the NTE. To maintain this identity and to avoid a night out being spoiled by USI, especially since effective action to address it is unlikely, women engage in emotion work which dampens their reactive emotions. Similarly, Brooks (2014) found that women whose drinks had been spiked were reluctant to adopt victimhood, minimized their experiences, and anticipated negative responses from others. Their drink-spiking narratives included a sense of ambivalence similar to that expressed by the term “uncomfortable.” Thus, the process of normalization may impact women's ability and willingness to identify and name the emotions they experience in response to USI.

Alternatively, it might be the case that these women did not experience the USI as significantly impactful and so the relatively mild term “uncomfortable” accurately reflects their emotional reactions (“not massive impact to me”). However, the following respondent's use of the emphasizing term, “deeply,” and reference to feeling “unsafe,” indicate that the term “uncomfortable” can denote significant and impactful experiences:

I have had things shouted at me and my female friends in [on] nights out, on the rare occasions I would go on one. Always by drunk men. It makes me deeply uncomfortable, and feel unsafe, and it has put me off going to places like that. I avoid venue hot spots in the city in the evening, even just passing by*.*

Clearly, the notion of discomfort encompasses a wide range and intensity of affects that future research could fruitfully explore.

### Anger

The third most commonly expressed feeling in the survey was anger (see Table 2). Bericat (2016, p. 492) describes anger as a primary emotion—“considered to be universal, physiological, of evolutionary relevance and biologically and neurologically innate.” He sees anger as

the node for an extensive family of emotions which range from simple annoyance or indignation, to rage or fury. It is most often stimulated by perceived or real insult, injustice, betrayal, lack of equality, obstacles to achievements, incompetence and physical aggression. (Bericat, 2016, p. 502)

Among our participants, anger was a common emotional reaction, sometimes experienced in combination with other feelings:

I was grabbed around the waist from behind and a male thrust his groin against my bottom. He had an erection at the time. My friend pushed him away, the barman confronted him and he became violent so he [barman] called the police. I’m very cautious when out now and prefer to stand where no-one can get behind me. I feel angry and upset that he thought that was ok.

A male approached me outside of a nightclub and tried to put his hand up my skirt I was so shocked, angry and upset he ran away from the scene.

These accounts suggest that anger can stem from, *inter alia*, feelings of injustice—“angry and upset that he thought that was ok.” Describing their feelings as anger suggests that respondents were confident that the USI was wrong, unreasonable, and indefensible because “[a]nger is a claim of injustice” (Winderman, 2019, p. 329). It could also suggest a sense that their rights and freedoms were ignored or, worse, trampled over. The (slightly) more frequent expression of anger rather than shame might reflect changing social norms (resulting from, for example, the #MeToo movement) which have partially destigmatized victimization, encouraged the expression of collective anger (Kay, 2020), and framed anger as a force for collective action (Ellefsen & Sandberg, 2022; Lewis et al., 2018; Winderman, 2019); anger is the current “emotional climate” (Bericat, 2016, p. 504) of GBV.

Moreover, women's social and personal history with anger is complex; “anger is perceived as incompatible with stereotypical prescriptions for women to be kind and caring” (van Breen & Barreto, 2022, p. 124); it is “the ultimate taboo in women's speech” (Kay, 2020, p. 48). Women are also at risk of being seen as *too* emotional and discounted as an “angry feminist” or an “angry Black woman.” Women may *feel* but not *express* anger due to these social norms and/or due to the risks of expressing anger; the quote above about intervention by a friend and a barman demonstrates that some men who sexually intrude respond aggressively to anger, increasing the risks to women.

Some respondents reported feeling anger on behalf of other women who were victimized. Indeed, engaging in the NTE can generate strong feelings of solidarity amongst women in recognition of the shared experiences of USI, as well as the opportunities for friendship and shared positive experiences (Gunby et al., 2020; McBride, 2020). The following extract gives a sense that the anger the respondents experienced emanated from strong feelings of the injustice of USI. Witnessing a man trying to convince the security staff that he was the boyfriend of a very drunk young woman, a respondent reported:

This absolutely infuriated me so after he had left I complained about him to the bouncer and explained everything that had happed [happened] previous. He was so understanding and kicked him out straight away. I am proud of myself for standing up for myself and another girl. It just makes me so incredibly upset because I just feel like he will try it again and maybe the next girl won’t have been so lucky (maybe not the right word choice here, I just didn’t know how to describe it—not being sexually harassed should not be down to “luck”).

### Shame

Anger was often felt in combination with other emotions. It was linked, for example, with shame:

I’ve been stood at a bar on a night out and had a man try to grab underneath my skirt, I was humiliated, embarrassed, disgusted and angry.

I’ve been harassed on multiple nights out by men, sometimes just cat calling or shouting/saying things other times forcing themselves on me to kiss me or put their hands on me, nobody else has ever helped in these situations and it made me feel ashamed, upset and angry.

Traditionally, a key feature of GBV and of its normalization has been its societal framing as shameful *for the victim-survivors*. This social norm means that victim-survivors, rather than perpetrators, carry stigma as a result of their victimization; it has operated as a form of policing to reinforce gendered norms, particularly for women, and particularly around sexuality. Conceiving shame as resulting from social norms fits with Bericat's (2016) categorization of it as a secondary emotion, in contrast to some scholars’, such as Kemper's (2006), consideration of it as a primary emotion. Bericat sees it as a secondary emotion because it is “socially and culturally conditioned” (2016, p. 492). However, it is no less powerful for this categorization; it is, he writes, “a very painful emotion, [felt] when we are rejected by or lose worth in the eyes of the other” (2016, p. 492). He argues that it is such an acute emotion because it reflects the fundamental human motive to maintain bonds. In considering feelings of shame in response to USI, we might consider these human bonds in terms of women's sense of equal status and legitimate occupation of space in the NTE. Their feelings of shame, mixed with humiliation (which is inherently generated by social interaction), and a sense that they are alone—“nobody else has ever helped”—suggest that bonds with others in the NTE are broken and, as a result, the women no longer “belong.”

The expression of emotions, alone and in combination, is complex and can be difficult to interpret. The simultaneous experience of the “very painful” (Bericat, 2016, p. 492) feelings of shame together with the apparently more empowered sense of anger, demonstrate this complexity. Feelings of anger may stem from a feminist consciousness or, in contrast, from non-feminist, conservative social norms about women protecting their “purity.” Some feelings might be expressed more frequently or more vociferously not simply because they are more commonly or more deeply felt but also because they comply with normative expectations about the expression of emotions by certain people in certain circumstances. Even though some emotions are considered primary, they are expressed in the context of changing social and political mores and sentiments which impact upon both individual and collective emotional experiences.

### Other Emotional Responses

Respondents expressed a range of other emotions. Many respondents used the term “upset” to describe their emotional reactions or described them as “horrible” or “awful.” Like “uncomfortable,” these are fairly elastic terms which refer to a range of emotions other than the primary emotions identified above. Upset is often felt in combination with other emotions, for example:

I’m a woman and I have been harassed for being a lesbian and kissing or dancing with women by men. From catcalling to physical. Makes me feel invalid and upset.

I’m a female, when I was 22 a man had pulled my top down and exposed my breasts. Felt very upset and vulnerable he made me feel insecure as there were hundreds of people in the club.

Some of our respondents reported their emotional reactions to USI as embodied responses:

Every time I’ve ever been in a club men are putting their hands on you, in crowded spaces a man totally unknown to us once tried to put his fingers inside my pants under my dress. When I spun round him and his mates were laughing. I feel physically sick.

I’ve been fingered^3^ by a stranger (man) on a night out in a bar. I assume because I was wearing a shirt dress. It made me feel horrible.

In contrast to extracts presented above which express a sense of injustice and anger, this last respondent's comments reflect a sense of self-blame—she takes responsibility for the abuser's behavior by referring to her clothing—or a sense that the man read her clothing in a way that reflected his entitlement to sexual access to her.

Some respondents’ embodied reaction to USI had the effect of shrinking their presence; it made them feel vulnerable or “small.”

They wouldn’t leave me alone tried to buy me drinks, trying to touch my body, tried to dance with me. It was a male and I’m a female. They were drunk. It made me feel small and it made me uncomfortable.

The following respondent's feelings of vulnerability stemmed from what she saw as heteronormative behavior whereby straight men routinely sexual objectify and intrude upon women:

My main impression of going to straight clubs is of men trying to dance close to me or touch me without me showing any interest in this. It makes me feel vulnerable. It stops me from wanting to go to straight nightclubs which means I get isolated from straight friends.

In the same way that online gendered abuse reduces women's access to and enjoyment of public space as well as their public voices (Lewis et al., 2017), USI in the NTE can reduce women's enjoyable occupation of and access to public space (Bows & Fileborn, 2022) and, in this case, her connection to straight friends.

A minority of respondents reported an embodied reaction of shock:

I was in a night club on the dance floor with a friend and a man tried to join in and we moved away he then followed and put his head into my chest and “motorboated” me (kind if blowing a raspberry and shaking his head). I was shocked so I just stood there for a moment before walking away and reported to staff, don’t know what happened to him after that. I just felt embarrassed and [it] ruined the rest of the night.

As discussed above, “freezing” is a relatively common reaction to unwanted sexual contact (Rape Crisis, no date). Women's behavior in response to USI varies, and has been well-considered elsewhere (see, e.g., Diaz-Fernandez & Evans, 2020; Fileborn, 2012; Graham et al., 2017; Gunby et al., 2020) but when their reaction is to “freeze,” they are unable to respond directly to the man who has intruded and, typically, their emotions are also frozen; both their emotions and behavior are shut down leaving them (temporarily) unable to react or to process emotions.

## Discussion

The survey data presented here show that women experience a range of emotions in response to USI, predominantly fear, discomfort, anger, and shame. We consider discomfort to be a term (like “upset”) that may mask a wide range of emotions that warrant further exploration. These relatively mild, ambivalent terms which are sometimes used to describe behavior that constitutes sexual assault may also reflect “the way in which women's accounts of sexual violence have been subject to denial and a culturally embedded suspicion of women's propensity to make ‘false allegations’ of sexual assault” (Brooks, 2014, p. 312).

Fear and anger are widely seen to be “primary” emotions in that they are universal and innate, while shame is considered by some to be a “secondary” emotion that stems from social and cultural conditioning (Bericat, 2016). They are all inherently dynamic and social in that they result from interactions with others. They are also social in that they are individual feelings that are both products of and help to (re)produce social, cultural, and political worlds. How, then do these emotions help develop a sociological analysis of USI?

Women's fear in response to USI, the most commonly reported emotion, indicates their sense that their personal safety was threatened and they anticipated further harm. Clearly, USI's ubiquity in the NTE does not minimize its significance. The fear expressed might stem not only from the immediate individual experience of USI but might also be shaped by the heightened attention to GBV and collective awareness of its nature and extent (see Pryor et al., 2023); in this way, it might reflect the contemporary “emotional climate” (Bericat, 2016, p. 504) around GBV. After all, emotions are not only spontaneous, ephemeral experiences; they also reflect personal and social legacies (Mason, 2018). Women's fear challenges the portrayal of the NTE as liberatory and hedonistic and shows this aspect of it is not equally available to all participants in the NTE. It also challenges the minimization of GBV which is part of its normalization. Moreover, fear of USI and other forms of GBV is significant because it leads women to modify their behavior and engage in “safety work” (Vera-Gray & Kelly, 2020) which restricts their freedom to engage in social, political, and economic aspects of society.

Women's anger in response to USI reveals several interesting issues. As an emotion that has been traditionally forbidden for women, and when expressed by them, has been pathologized, anger is coded as masculine. However, the recent increase in attention to GBV has been accompanied by a growing anger, if not rage, expressed by women around the globe (see, e.g., Bhandare, 2019; Bloomberg News, 2022; Kay, 2020; Topping, 2021; Winderman, 2019). Anger expresses the injustice of GBV, and powerfully asserts that the angry person is not responsible for the injustice, that responsibility lies elsewhere. This is reflected in Honkatukia et al.'s (2023) finding of “a significant shift in young women's sexual citizenship, presenting them[selves] now as citizens who are without questioning entitled to protection from adverse sexual experiences” (Honkatukia et al., 2023, p. 1395). Our respondents’ reports of anger may similarly reflect that anger is now a more acceptable emotion, indeed may be the *obvious,* even *required* response to endemic GBV; (some) women are now allowed and encouraged to express anger in a way previous generations have not been. However, anger remains a socially prohibited emotion for some; Winderman (2019, p. 329) notes “anger waxes and wanes through public life along raced, gendered, and classed lines that too often elevate the righteous expression of privileged anger while ignoring or silencing the anger of those most marginalized.” Moreover, Orgrad and Gill's (2019, p. 601) analysis of female rage in public life warns that the anger itself may become the story rather than the injustice it responds to because “even when unleased, this anger continues to be carefully regulated so as not to exceed the ‘safe’ level allowed by a patriarchal system.” Clearly, even though it is widely considered to be a primary emotion, anger is also subject to social and cultural influences which determine norms about its expression.

Even if the public conversation about GBV *is* changing perceptions and attitudes, women continue to carry shame in relation to USI; expressions of shame were almost as common as expressions of anger in our dataset. Inscribing GBV as shameful *for the victim-survivor* rather than the perpetrator, has been a fundamental aspect of the maintenance of men's violence; “[s]hame operates as a gendered set of self-regulatory practices, which are also practices of male power in individual women's lives” (Baker, 2013, p. 145). Shame and anger may seem to be contradictory emotions; if women believe an injustice has occurred (anger), why would they also feel shame, which suggests that they are taking some responsibility for their experience? The answer lies in the social and cultural nature of these emotions for women who experience USI. Traditionally, women have been held responsible for their own victimization but there is a contemporary shift towards re-framing USI as an injustice and holding abusers to account. Perhaps the apparently contradictory mixture of emotions reported by women in our study reflects both the traditional and contemporary framings of USI, highlighting the “emotional climate” (Bericat, 2016, p. 504) that characterizes this moment in the history of GBV.

Despite the feelings of anger felt by some women in response to USI, it is worth noting that relatively few respondents said that they had stopped engaging in the NTE because of such experiences. The NTE continues to be a key site of social interaction in most cities and towns; for most women, the threat of experiencing USI does not prevent them from participating. This suggests that the positive aspects of the NTE outweigh the risks, or that they have few alternative options for socializing that are free of USI.

This study has used qualitative survey data from a sample of women in the UK. A more in-depth study about emotional responses could usefully explore the relationship between anger and fear in relation to USI as well as the relationship between anger and shame. A longitudinal approach would help reveal the changing nature of the emotional landscapes and societal framing of USI. Further research could also capture the emotions as close to the event as possible through, for example, participant diaries or ethnography, to identify which emotions endure, and which are considered normative emotional responses to USI. Women's experiences are shaped as well by class, race, sexuality, disability, age, and other demographic variables, consideration of which is beyond the scope of this article but warrants further attention. Given the very limited body of work about the orientations, feelings, attitudes, and behaviors of the men who sexually intrude on women in the NTE, future research would also usefully address this aspect.

This study shows that women experience USI in the NTE as a frightening, shameful injustice. The continuing, changing public conversation about GBV (Kay, 2020) is likely to impact both women's individual and collective responses to USI in the future. Whether these changing emotions impact the men who intrude and their behavior remains to be seen.

## Notes

1. “When we say the **night-time economy**, we mean activity or events that happen between 6 pm and 6 am usually in bars, pubs, clubs, restaurants, music venues, cinemas.” “When we say **sexual harassment**, we mean unwanted conduct of a sexual nature. This can include whistling, sexual comments or ‘catcalling’, as well as non-verbal acts that include looking someone up and down, making sexual gestures, asking for sexual favours and online or image-based behaviors such as upskirting. We also include unwanted physical contact of a sexual nature.”

2. Data are presented verbatim, complete with typos and syntax errors, although, where appropriate, we provide in square brackets our assumption about the intended word of spelling, or clarifying text.

3. “Fingered” is a British colloquialism which refers to digital touching and/or penetration of the vagina.
